# Current Insights into the Roles of LncRNAs and CircRNAs in Pulpitis: A Narrative Review

**DOI:** 10.3390/ijms252413603

**Published:** 2024-12-19

**Authors:** Dulce Martha Fuchen-Ramos, Ana Gabriela Leija-Montoya, Javier González-Ramírez, Mario Isiordia-Espinoza, Fernando García-Arévalo, Viviana Pitones-Rubio, Carlos Olvera-Sandoval, Isis Mateos-Corral, Nicolás Serafín-Higuera

**Affiliations:** 1 Facultad de Odontología Mexicali, Centro de Ciencias de la Salud Mexicali, Universidad Autónoma de Baja California, Zotoluca s/n, Fracc. Calafia, Mexicali 21040, BC, Mexico; fuchend@uabc.edu.mx (D.M.F.-R.); fgarcia67@uabc.edu.mx (F.G.-A.); viviana.pitones@uabc.edu.mx (V.P.-R.); isis.mateos@uabc.edu.mx (I.M.-C.); 2 Facultad de Medicina Mexicali, Universidad Autónoma de Baja California, Centro Cívico, Mexicali 21000, BC, Mexico; gabriela.leija@uabc.edu.mx (A.G.L.-M.); olvera.carlos@uabc.edu.mx (C.O.-S.); 3 Facultad de Enfermería, Universidad Autónoma de Baja California, Av. Álvaro Obregón y Calle “G” S/N, Col. Nueva, Mexicali 21100, BC, Mexico; javier.gonzalez.ramirez@uabc.edu.mx; 4 Departamento de Clínicas, División de Ciencias Biomédicas, Centro Universitario de Los Altos, Universidad de Guadalajara, Av. Rafael Casillas Aceves 1200, Tepatitlán de Morelos 47600, JAL, Mexico; mario.isiordia162@yahoo.com

**Keywords:** long non-coding RNA, lncRNA, circular RNA, circRNA, pulpitis, inflammation

## Abstract

Pulpitis, an inflammation of the dental pulp, is generated by bacterial invasion through different ways as caries. In the establishment and development of this disease, different biological processes are involved. Long non-coding RNAs (lncRNAs) and circular RNAs (circRNAs) are transcripts with regulatory capacity participating in different biological functions and have been implicated in different diseases. The aim of this narrative review is to critically analyze available evidence on the biological role of lncRNAs and circRNAs in pulpitis and discuss possible new research prospects. LncRNAs and circRNAs involved in pulpitis were explored, addressing their expression, molecular mechanisms, targets and biological effects studied in animal and in vitro models, as well as in studies in human patients. LncRNAs and circRNAs are emerging as key regulators of diverse biological functions in pulpitis including apoptosis, proliferation, differentiation, oxidative stress, autophagy, ferroptosis, inflammation and immune response. The molecular mechanisms performed by these non-coding RNAs (ncRNAs) involved interactions with miRNAs and the formation of regulatory networks in the context of pulpitis. Further studies more deeply analyzing the participation of lncRNAs and circRNAs in pulpitis will reveal the potential applications of these ncRNAs as biomarkers or their use in therapeutic strategies in pulp inflammation.

## 1. Introduction

Dental pulp is composed of connective tissue, nerve fibers, blood vessels and various cell types that perform specialized functions to sustain the tooth’s biological and physiological vitality [1]. Pulpitis, an inflammation of the dental pulp, is primarily triggered by bacterial invasion through caries, trauma, dentinal cracks and exposure via the main apical foramen or dentinal tubules. Once these pathogens breach the pulp’s defenses, immune cells such as macrophages, dendritic cells, odontoblasts and endothelial cells recognize pathogen-associated molecular patterns (PAMPs) through Toll-like receptors (TLRs), initiating a robust immune response [2,3,4]. These cells release cytokines and chemokines, small immune mediators that promote cell growth, differentiation and chemotaxis, that are central to the inflammatory process [4]. Adaptive immune responses may also emerge in prolonged pulpitis, with T cells and B cells releasing antibodies and additional cytokines to neutralize bacterial toxins and facilitate phagocytosis [5]. Reactive oxygen species (ROS) and other reactive species are generated as part of the inflammatory response, and ROS levels are increased in human pulpitis and pulp cells in cultures treated with bacterial molecules. While controlled basal ROS levels can aid immune cell function, activate pulp cells, including stem cells essential for repair, and support signaling for cellular activities like gene expression, proliferation and migration, elevated ROS levels can harm host cells [6]. Understanding these pathways offers valuable insights into targeted therapeutic approaches that could improve pulpitis management and promote tissue repair [5].

The current diagnostic guidelines designate a vital pulp as normal, reversible pulpitis or irreversible pulpitis (which could be symptomatic or asymptomatic), and the designation is based upon subjective and objective findings considering pain history and proper clinical testing [7,8]. Limitations of this classification system of pulp disease have been addressed [7,9], and a relatively new classification of pulp inflammation has been reported suggesting different stages of pulpitis (initial, medium, moderate and severe pulpitis) [9].

On the other hand, non-coding RNAs (ncRNAs) are a varied group of transcripts that classically do not encode proteins and differ in size, structure and functions. NcRNAs, such as microRNAs (miRNAs), long non-coding RNAs (lncRNAs) and circular RNAs (circRNAs), can regulate different biological functions, including inflammation and immune function [10]. Roles of miRNAs in pulpitis have been reviewed previously [11] and will not be revised in the present work; functions and regulatory molecular mechanisms of lncRNAs and circRNAs have recently begun to be analyzed in pulpitis.

### 1.1. LncRNAs

LncRNAs are transcripts characterized by more than 200 nucleotides (200 nt) in length that lack functional open reading frames (ORFs) and do not encode proteins [12]. Despite the classic definition of lncRNAs, it has been reported that they are more than 500 nt length, and some transcripts annotated as lncRNAs have turned out to encode micropeptides [13,14,15]. Regarding its biogenesis, lncRNAs are mainly generated by RNA polymerase (Pol) II and undergo 5′ capping, splicing and 3′ polyadenylation. In addition, lncRNAs that are not polyadenylated or capped are transcribed by Pol I or Pol III [13]. As general features, lncRNAs present linage-specific and tissue-specific patterns, spatiotemporal regulation and subcellular localization, and their expression is influenced by environmental changes. Also, with some exceptions, lncRNAs have lower expression compared to protein-coding transcripts (mRNAs) [13].

Currently, it has been established that lncRNAs have great relevance in transcription regulation [16], achieving their functions either in cis or trans, regulating the expression of neighboring or distant genes through different mechanisms acting as (1) guide lncRNAs, through relocalization of regulatory factors, (2) scaffold lncRNAs, inducing the formation of ribonucleoprotein (RNP) complexes, (3) decoy lncRNAs, removing regulatory factors bound to the genome, (4) sponge lncRNAs, by sequestering miRNA, (5) miRNA precursors, in which the lncRNAs are processed into mature miRNA, and (6) chromatin loops, in which lncRNAs from regulatory regions produce a chromatin looping by recruiting chromatin-modifying factors to regulate distant genes [17,18]. In summary, lncRNAs can regulate biological processes such as genetic imprinting, chromatin modification, RNA processing and protein translation.

In fact, lncRNAs have emerged as potential key players of the inflammatory response and appear to be an integral component of inflammation and immune response [18,19], acting as serious regulators of it by affecting the production of immunological mediators, such as cytokines and chemokines. In addition, lncRNAs contribute to the necessary hemostasis of the immune reaction; some lncRNAs intensify inflammatory signals while others act as inhibitors of the inflammatory response [20]. In the innate immune response, lncRNAs are currently involved in the conservation of hematopoietic stem cells, direct differentiation and programmed cell death of myeloid cells and promotion of the activation of monocytes, macrophages and dendritic cells [20]. Regarding adaptive immunity, lncRNAs participate in regulatory circuits that dictate the development, activation and differentiation of lymphocytes. Also, lncRNAs cooperate with key transcription factors responsible for T cell polarization and improve the production of cytokines. As a result, lncRNAs have an important role in cellular and humoral response [21]. Considering this plethora of functions, lncRNAs have been analyzed in different inflammatory diseases including pulpitis.

### 1.2. CircRNAs

CircRNAs are a type of single-stranded RNA characterized by a covalently closed-loop structure [22]. The unique structures of circRNAs provide resistance to exonucleases and increased stability compared to linear mRNAs. Also, their sequences are conservative to some extent. CircRNA biogenesis is a process that is not completely understood, and it is specific to each cell type. Due to their evolutionarily conserved structure and cell-specific expression profiles, circRNAs regulate themselves independently of their linear transcripts [23]. Most circRNAs are produced by back-splicing, a spliceosome-dependent process, where a splice donor site downstream connects to an upstream splice acceptor site, producing the circularized formation that is facilitated by RNA-binding proteins and the intron sequences that flank the exons that will conform to the circRNA. According to their sequence, circRNAs are classified into four groups: (1) intronic circRNAs (ciRNAs), (2) exonic circRNAs (EcircRNAs), (3) exon–intron circRNAs (EIciRNAs) and (4) tRNA intronic circular RNAs [23,24].

CircRNAs are associated with neoplastic, respiratory, metabolic, musculoskeletal, cardiovascular and renal diseases [24,25]. Moreover, many studies have reported the role of circRNAs in the initiation, development and resolution of inflammatory diseases, performing different processes such as the regulation of RNA transcription and protein translation, the modification of DNA structure and, the most studied mechanism, the sequestration of miRNAs by circRNA sponges [23].

Even though some circRNAs are located in the nucleus (ciRNAs and EIciRNAS), most circRNAs are citoplasmatic. Also, the presence of abundant circRNAs in extracellular vesicles has been demonstrated, possibly as a suggested mechanism to remove accumulated intracellular circRNAs [23]. The extracellular vesicles are lipid bilayer-enclosed nanosized vesicles derived from various cell types, presented in extracellular space and different biological fluids as mediators of intercellular communication, capable of carrying transmembrane molecules and internal cargos such as nucleic acids, proteins and lipids. The circRNAs present selective packaging into extracellular vesicles and can be delivered to neighboring and distant recipient cells to regulate different processes [26]. Extracellular vesicles, particularly exosomes, as carriers of circRNAs, have been studied as a promising mechanism of diagnosis and treatment [24].

This narrative review arose from the observation that ncRNAs involved in different diseases have been analyzed and discussed, including the examination of approaches to therapeutic and diagnostic applications [27,28,29]. In this sense, different recent reviews have addressed the importance of lncRNAs and circRNAs in the regulation of biological processes in dental pulp stem cells (DPSCs), incorporating discussion about their possible applications [30,31,32,33]. However, to the best of our knowledge, a review focused on the lncRNAs and circRNAs involved in pulpitis has not been previously published. The aim of this work is to critically analyze available evidence on the biological contribution of lncRNAs and circRNAs in pulpitis and discuss possible new research prospects. Emergent evidence of validated lncRNAs and circRNAs, described molecular mechanisms, the reported targets and the biological effects analyzed in cell culture and animal models of pulp inflammation, as well as in samples of patients with pulpitis, have been summarized and discussed.

## 2. Methodology

A literature search was performed between May and October of 2024. No time restrictions were imposed as a search criterion. The databases PUBMED, SCOPUS and Web of Science were utilized, using a combination of search terms: long non-coding RNA, lncRNA, long non-coding RNAs, lncRNAs, pulpitis, pulp inflammation, dental pulp, circular RNA, circRNA, circular RNAs and circRNAs. Inclusion criteria were use of the English language and publications involving lncRNAs and circRNAs in human pulpitis, in vitro or animal models mimicking the characteristics of pulpitis. Emphasis was placed on the identification of lncRNAs and circRNAs with validated expression by quantitative real-time polymerase chain reaction (qRT-PCR), biological processes modulated by these ncRNAs, experimental analysis of the involved molecular mechanisms and molecular targets.

## 3. LncRNAs and Pulpitis

Differential expression of lncRNAs has been addressed in samples of human pulpitis [34,35,36,37]. Additionally, models mimic some characteristics of pulpitis such as experimental pulpitis and pulp cell cultures treated with lipopolysaccharide (LPS) [38,39,40], and cytokines [41] have been used to analyze the expression and biological functions of lncRNAs.

The expression of lncRNAs has been analyzed in the dental pulp of patients with pulpitis showing notable differences in the number of lncRNAs differentially expressed in inflamed dental pulp as compared with normal pulp [34,35,36,37,39]. Previously, an analysis of lncRNA expression by microarray identified 752 lncRNAs differentially expressed in inflamed dental pulp samples of patients with irreversible pulpitis compared with healthy pulp tissues: 338 lncRNAs were upregulated and 414 lncRNAs were downregulated [34]. An independent report analyzed these same data, identifying 278 lncRNAs differentially expressed (138 upregulated and 136 downregulated) [35]. However, two independent bioinformatic analyses of these data showed 90 and 27 lncRNAs which were differentially expressed in samples of pulpitis compared with healthy pulp samples [36,37]; these apparent discrepancies in the number of lncRNAs expressed differentially could be due to differences in the data processing. In addition, an independent study showed that 81 lncRNAs were differentially expressed in inflamed human dental pulp in comparison to healthy pulp, with upregulation of 32 lncRNAs and downregulation of 49 lncRNAs [39]. Together, these reports indicate significant changes in expression of lncRNAs in pulpitis to different extents; it is possible that these apparent discrepancies could be due to differences in data analyses or variability in the patient samples.

The validation by qRT-PCR of these data showed the increased expression of different lncRNAs, such as RP4-754E20_A.5, XLOC_01234, XLOC_000283, LINC02618, LINC02828, LINC01926, LINC01094, LINC02705, LINC00278, LINC01724, LINC01857, ANKRD44-IT1, IL10RB-DT, LINC02148, LINC02123 and LINC01665, as well as the reduced expression of LINC00475, ADAMTS9-AS2 and LINC00290 in samples with pulpitis compared with control samples of healthy pulp [34,36,37,42]; however, the biological functions of these lncRNAs in pulpitis or the involved molecular mechanisms were not explored experimentally.

Bioinformatic analyses of different reports have indicated the participation of lncRNAs in regulatory axes modulating diverse biological functions and molecular mechanisms in pulpitis. For example, it was suggested that lncRNAs such as XIST, MIR155HG and LINC00630 could be part of competing endogenous RNA (ceRNA) regulatory networks in human pulpitis [35]; however, the expression validation and involved molecular mechanisms remain to be determined experimentally. In these networks, lncRNAs can interact with miRNAs, regulating each other and the expression of downstream target genes, indirectly resulting in the modulation of biological processes in pulpitis [43]. A study suggested that ferroptosis can be involved in pulpitis and that this iron-dependent regulated cell death could be modulated by lncRNAs [44]. Ferroptosis, generated by lipid peroxidation, which causes damage of the membrane [45], is a process that could regulate immune cell functions, and it has been related to inflammation [46]. In silico analysis identified 201 differentially expressed lncRNAs related to ferroptosis in pulpitis [44]. Validation showed decreased expression of lncRNAs AL583810.1, AC068888.1, AC125257.1 and LINC00943, while increased expression of AC106897.1 was detected in pulpitis [44]. Regulation of ferroptosis and the mechanisms performed by these lncRNAs were not addressed. Additional studies are necessary since the impact of ferroptosis in pulpitis is widely unknown.

Another biological process closely associated with pulpitis is autophagy [47]. This phenomenon allows the maintenance of cellular homeostasis, leading to the degradation and recycling of organelles and proteins, and it was reported that lncRNAs could be involved in modulation of autophagy during pulp inflammation [47,48]. Bioinformatics analysis indicated 210 differentially expressed lncRNAs related to autophagy in pulpitis. The validation showed the increased expression of lncRNAs HCP5 and AC112496.1 and diminished expression of AC145207.5, TMEM161B-DT, LAMTOR5-AS1, DLX6-AS1, ZSWIM8-AS1, AC099850.1 and FENDRR in pulpitis [48]; however, the molecular mechanisms regulating autophagy performed by these lncRNAs were not established experimentally.

Taken together, these in silico analyses suggested that lncRNAs could modulate the expression of miRNAs and regulate processes such as ferroptosis and autophagia in pulpitis, as they are part of competing endogenous RNA (ceRNA) regulatory networks. However, experimental studies are necessary to establish the interaction between the possible lncRNAs, miRNAs and mRNAs forming these networks and to determine the biological effects.

Since inflammation and immune response are paramount processes in pulpitis, the contribution of lncRNAs to these biological mechanisms has been addressed. In this sense, it was reported that lncRNAs are involved in distinct immunological pathways. This was determined by analyzing in silico databases of differentially expressed lncRNAs in samples of human pulpitis. The results identified 38 immune-related lncRNAs [49]. The experimental validation of these data showed the increased expression in pulpitis of the following immune-related lncRNAs: LINC02828, IL10RB-DT, LINC01094 and ANKRD44-IT1 [49]. The molecular mechanisms in which these lncRNAs and their possible targets are involved remain to be determined experimentally.

**Figure 1:**
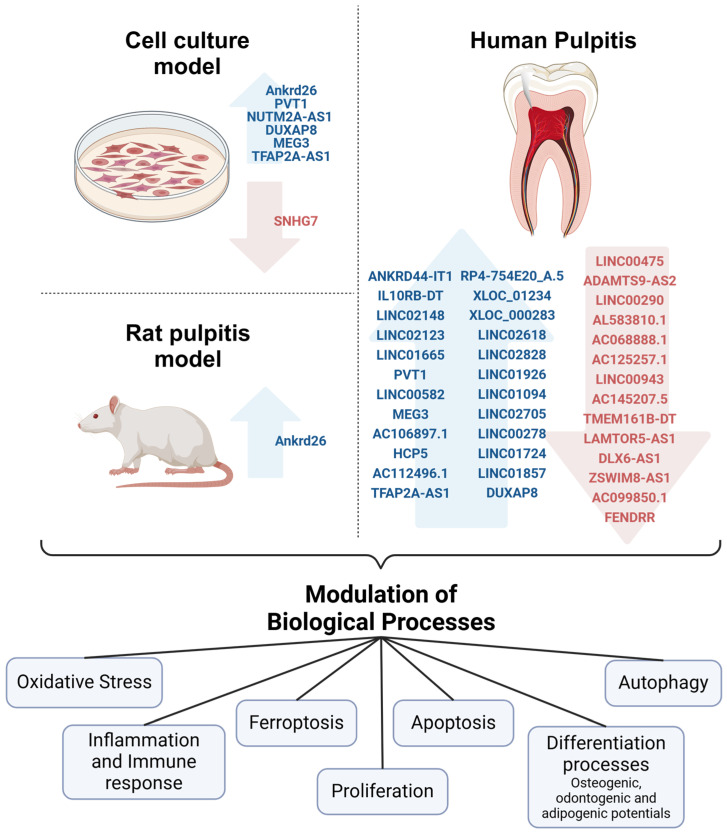
LncRNAs can modulate different biological processes in the context of pulpitis. LncRNAs expressed differentially and validated by qRT-PCR in pulpitis, including analyses in vitro and animal models, as well as studies in patients, are indicated. More lncRNAs have been analyzed in pulpitis; however, expression was not validated, and they were not included in this figure. Blue arrows indicate increased expression. Red arrows indicate decreased expression. The biological processes possibly regulated by validated lncRNAs in human pulpitis, as well as in models resembling some characteristics of this disease, are shown. Created with BioRender.com.

Thus, lncRNAs could be involved in the modulation of different cell processes in pulpitis (Figure 1). In this sense, some molecular mechanisms by which some lncRNAs can modulate different biological functions in the context of pulpitis have been described and analyzed experimentally and are discussed in the next sections.

### 3.1. LncRNA PVT1

The expression of the lncRNA PVT1 is increased in samples of human pulpitis as compared with control samples [36]. In accordance, augmented levels of PVT1 expression have been reported in the saliva of patients with pulpitis [50]. Interestingly, in silico evaluation suggested that the lncRNA PVT1 can act as a sponge to miR-455-5p. Thus, the increased expression of PVT1 in inflamed pulp promotes downregulation of miR-455-5p, resulting in upregulation of SOCS3 (suppressor of cytokine signaling 3) and PLXNC1 (plexin C1) mRNAs, which can be targets of miR-455-5p, and it was suggested that these genes could be involved in the promotion of inflammation [36]. However, this remains to be established experimentally.

Additionally, it was reported that the expression of PVT1 is increased in a model of pulpitis in vitro using LPS-treated human dental pulp cells (LPS-hDPCs). It was suggested that the lncRNA PVT1 can promote apoptosis, secretion of TNF (tumor necrosis factor)-α, TNF-β and IL (interleukin)-6 and a reduction in cell viability in LPS-hDPCs. The molecular mechanism involved the binding of PVT1 to miR-128-3p, resulting in the reduced expression of this miRNA [50]. Additional miRNA targets of PVT1 should be established as well as downstream indirect targets regulated by these miRNAs to explain more deeply the molecular mechanisms modulating the biological effects reported. Moreover, the analysis of the modulation of inflammation and apoptosis by PVT1 in animal models of pulpitis could determine the potential of PVT1 as a therapeutic target.

### 3.2. LncRNA MEG3

In situ hybridization and qRT-PCR showed that the gene expression of lncRNA MEG3 is increased in human inflamed dental pulp in comparison with normal pulp [39]. In vitro, this lncRNA is upregulated in the nuclei and cytoplasms of LPS-hDPCs [39], suggesting potential biological activity in different cell compartments. Moreover, lncRNA MEG3 can bind directly or indirectly to 209 proteins in hDPCs, and these proteins have been related to cell components such as extracellular vesicles as well as p38/MAPK (mitogen-activated protein kinase) and Wnt (Wnt family member)/β-catenin signaling pathways [39]. Thus, it was suggested that this lncRNA could be a cargo molecule in extracellular vesicles; however, this was not demonstrated, although previous studies have indicated that MEG3 can be carried by vesicles and delivered to cells generating different biological effects [51,52]. LncRNA MEG3 can promote secretion of IL-6, IL-1β and TNF-α in LPS-hDPCs, possibly involving the p38/MAPK signaling pathway. Also, this lncRNA negatively regulated the osteogenic/odontogenic differentiation of hDPCs in vitro through the Wnt/β-catenin signaling pathway [39]. However, the mechanisms of how MEG3 interacts/modulates these signaling pathways were not determined. Thus, lncRNA MEG3 appears to be important in the regulation of biological effects during pulpitis. Further studies in animal models or patients could support the significance of this lncRNA in pulp inflammation and its possible applications as a therapeutic target.

### 3.3. LncRNA NUTM2A-AS1

Analyses of microarray data showed increased expression of NUTM2A-AS1 in human pulpitis [36]; however, expression was not validated. hDPCs showed increased expression of NUTM2A-AS1 when treated with LPS. It was suggested that NUTM2A-AS1 overexpression is important in the promotion of inflammation and apoptosis observed in this in vitro model since the knockdown of this lncRNA decreased production of IL-8 and IL-6, promoted the reduction in positive cells to annexin-V and propidium iodide and diminished the expression of proapoptotic proteins Bax (BCL2-associated X, apoptosis regulator) and C-casp 3(cleaved caspase 3) in LPS-hDPCs [40]. The molecular mechanism involved the interaction between the miRNA let-7c-5p and the 3′-UTR (untranslated region) of HMGB1 (high mobility group box), with increased expression of let-7c-5p, resulting in the decreased expression of HMGB1. Interestingly, apoptotic and inflammatory processes are promoted by HMGB1. Additionally, it was suggested that NUTM2A-AS1 can interact with let-7c-5p and reduce its expression, functioning as a competitive endogenous RNA. Thus, overexpression of the lncRNA NUTM2A-AS1 stimulates the expression of HMGB1, causing apoptosis and inflammation in the in vitro model to study pulpitis [40]. Analysis in patients could establish the relevance of these molecular mechanisms in more complex systems and support the potential as a therapeutic target of this lncRNA.

### 3.4. LncRNA Ankrd26

Differential expression of lncRNAs was founded in an experimental pulpitis model in rats. Although the amount of lncRNAs expressed differentially in this model was not specified, the lncRNA Ankrd26 was more increased in the model than in healthy control pulp [38]. In vitro analyses suggested that lncRNA Ankrd26 is produced by rat DPSCs (rDPSCs) and delivered inside of extracellular vesicles, which are taken up by cells, such as MSCs (mesenchymal stem cells), resulting in migration and osteogenic differentiation [38]. Mechanistically, lncRNA Ankrd26 induces these biological effects in MSCs, binding to miR-150 and reducing the levels of this miRNA [38]. Since the mir-150 can bind to the 3′-UTR of TLR4, reducing its expression, and the TLR4 downexpression results in decreased migration and diminished expression levels of osteogenic differentiation markers (such as OCN (osteocalcin), OPN (osteopontin) and RUNX2 (RUNX family transcription factor)), the augmented levels of lncRNA Ankrd26 promote increased expression of TLR4 in MSCs [38]. It was suggested that this lncRNA could participate in the processes of dental pulp restoration and repair; however, further analyses in more complex models should determine the actual contribution of lncRNA-Ankrd26 in these biological functions. Additionally, since the report used a rat model, studies in human cells or patient samples should be undertaken to determine the extent to which what was observed in the animal model aligns with the response in humans.

### 3.5. LncRNA DUXAP8

DUXAP8 is mainly found in the hDPC cytoplasm and is highly expressed in tissues of pulpitis from patients and in the in vitro pulpitis model using LPS-hDPCs [53]. In this model, the LPS treatment promotes oxidative stress (with decreased activity of superoxide dismutase, an enzyme considered with antioxidant capacity, and increased level of malondialdehyde, a product of lipid peroxidation used as stress indicator [53,54]), the production of IL-6 and IL-1β and apoptosis, with increased expression of Bax and reduced production of Cyclin-D1. These biological effects were related to the increased expression of DUXAP8, since the experimentally induced downregulation of this lncRNA in LPS-hDPCs partially reversed these effects [53]. Mechanistically, DUXAP8 binds to miR-18b-5p, resulting in downregulation of this miRNA expression. Additionally, miR-18b-5p can interact with the 3′-UTR of HIF3A (hypoxia-inducible factor 3A), promoting reduced HIF3A expression [53]. Thus, increased expression of DUXAP8 can promote apoptosis, inflammation and oxidative stress in pulpitis through negative regulation of miR-18b-5p, generating augmented levels of HIF3A [53]. Identification of additional targets of DUXAP8 establishing ceRNA networks could help to more clearly explain the different biological effects modulated by this lncRNA to propose possible applications in the treatment of pulpitis.

### 3.6. LncRNA SNHG7

Expression of SNHG7 is increased during osteogenic/odontogenic differentiation of hDPSCs (human dental pulp stem cells), and it was suggested that it promotes this process [55]. In an in vitro model resembling inflammatory conditions, the treatment of hDPSCs with TNF-α impaired their capacity for osteogenic/odontogenic differentiation, inducing reduced calcium deposition, lower ALP (alkaline phosphatase) activity and diminished mRNA expression of differentiation biomarkers such as IBSP (integrin-binding sialoprotein), DMP1 (dentin matrix acidic phosphoprotein 1), DSPP (dentin sialophosphoprotein), RUNX2 and OPN [41]. Also, the TNF treatment decreased the expression of SHNG7. However, the induced overexpression of SNHG7 in TNF-α-treated hDPSCs under conditions of differentiation reversed the indicated effects, indicating that SHNG7 promotes hDPSC osteogenic/odontogenic differentiation [41]. The molecular mechanism implicated the binding of this lncRNA with mir-6512-3p, resulting in reduced expression of the miRNA. Thus, TNF-α inhibits the osteogenic/odontogenic differentiation capacity of hDPSCs, promoting the diminished expression of SHNG7, which generates increased expression of mir-6512-3p [41]; however, direct targets of this miRNA were not determined. Analyses of the expression of SNHG7 in human pulpitis or animal models could determine its real contribution in more complex systems. Additional miRNAs regulated by SHNG7 and identification of indirect downstream targets would permit us to understand the role of this lncRNA in the modulation of differentiation in the context of pulpitis.

### 3.7. LncRNA TFAP2A-AS1

Pulp tissues from patients with pulpitis showed more expression of TFAP2A-AS1 than healthy pulps. In an inflammatory model, LPS treatment of hDPSCs (LPS-hDPSCs) increased the expression of this lncRNA, augmented levels of apoptosis and resulted in the secretion of TNF-α, IL-1β and IL-6 [56]. However, the induced downexpression of TFAP2A-AS1 in LPS-hDPSCs reversed these biological effects. It was suggested that TFAP2A-AS1 can interact with miRNA mir-32-5p, inducing downexpression [56]. Thus, the increased expression of TFAP2A-AS1 in pulpitis could promote inflammation and apoptosis of hDPSCs by downregulating the miRNA mir-32-5p. However, downstream targets regulated by this miRNA were not described or analyzed. Additionally, increased expression of TFAP2A-AS1 impaired the capacity of osteogenic/odontogenic differentiation of hDPSCs, showed by decreased expression of the markers DSPP and DMP1 as well as reduced levels of ALP activity [56], although the involved mechanism was not described. More studies should establish the mechanisms of how TFAP2A-AS1 can modulate different cell processes in pulpitis to determine the potential in therapeutic applications.

### 3.8. LncRNA LINC00582

The expression of LINC00582 was augmented in samples of patients with pulpitis compared with healthy pulp [42]. In vitro analyses have shown that hDPCs treated with LPS did not increase expression of this lncRNA [42]. Furthermore, artificially induced overexpression of LINC00582 in LPS-hDPCs did not reverse or exacerbate the effects generated by the LPS treatment such as reduced viability or increased expression of proinflammatory cytokines (IL-6 and IL-8) [42]. Thus, it was suggested that LINC00582 does not modulate biological effects in hDPCs under inflammatory conditions. Interestingly, in silico analyses suggested regulation of tissue-infiltrating B cells by lncRNAs in pulpitis. The induced overexpression of LINC00582 in a cell line of B cells promoted an increase in migration, proliferation and invasion [42], although the implicated mechanisms were not determined. It was suggested that this lncRNA could modulate the inflammatory microenvironment through the regulation of immune cells. Further studies are necessary to understand the mechanisms that shape the generated microenvironment in pulpitis since the expression of lncRNAs modulating functions in different cell populations of pulp could be involved.

### 3.9. LncRNA FTX

FTX is an lncRNA expressed by hDPSCs and localized in the cytoplasm and nucleus [57]. In vitro analyses showed that overexpression of this lncRNA inhibited proliferation as well as odontogenic and adipogenic differentiation capacities of hDPSCs treated with LPS. Mechanistically, the OCT4 (POU class 5 homeobox 1) protein can bind the promoter region of FTX, inhibiting its expression; thus, the augmented expression of OCT4 induces increased differentiation potential and proliferation [57]. Interestingly, FTX overexpression can inhibit the expression of the pluripotent transcription factors OCT4, SOX2 (SRY-box transcription factor 2) and c-MYC (MYC proto-oncogene, BHLH transcription factor) in LPS-hDPSCs by a molecular mechanism that is not yet determined, which could be related to impaired differentiation capacity under inflammatory conditions [57]. The expression of this lncRNA remains to be evaluated in human pulpitis. Studies in animal models or more complex systems will determine the molecular mechanisms involved in this FTX/OCT4 feedback mechanism and its real contribution in pulpitis to establish potential applications.

The molecular mechanisms carried out by these lncRNAs are summarized in Table 1.

## 4. CircRNAs and Pulpitis

**Figure 2:**
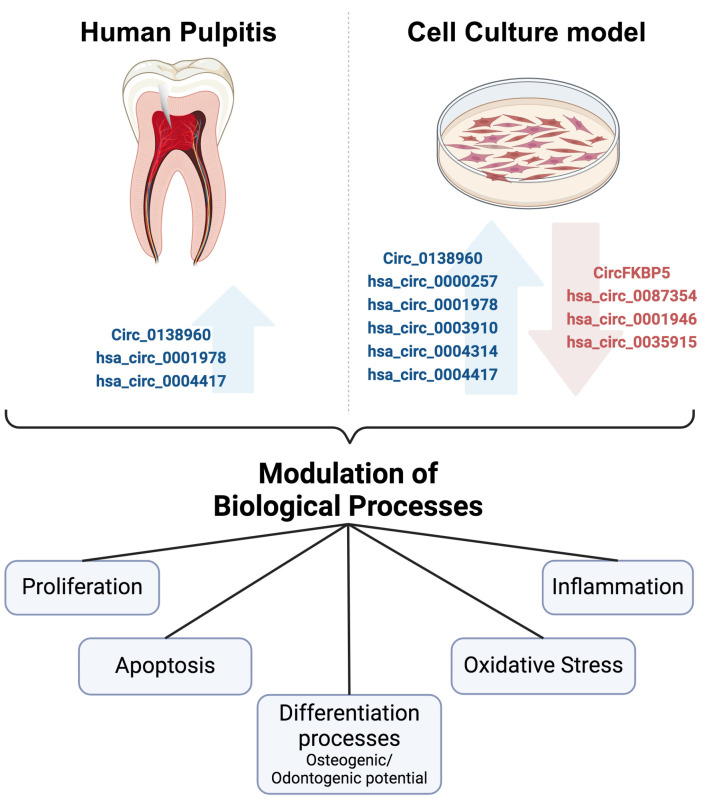
CircRNAs can modulate diverse biological processes in the context of pulpitis. CircRNAs expressed differentially and validated by qRT-PCR in human pulpitis and in vitro models, resembling some characteristics of this disease, are indicated. Blue arrows indicate increased expression. Red arrow indicates decreased expression. Additionally, these circRNAs could be involved in regulation of different cellular processes. Created with BioRender.com.

Analyses of the expression of circRNAs in samples of patients with pulpitis are very scarce. Furthermore, circRNAs have been analyzed in vitro using hDPCs and hDPSCs treated with different molecules, for example, LPS or cytokines, to resemble some aspects of an inflammatory environment such as that occurring in pulpitis. These studies have addressed the role of circRNAs in the modulation of diverse cellular processes (Figure 2) and the possible molecular mechanisms involved (Table 1).

### 4.1. Circ_0138960

It was reported that the expression of Circ_0138960 is increased in tissues of patients with pulpitis as compared with controls [58]. Also, the expression of this circRNA is augmented in a model of pulpitis using LPS-hDPCs. Functionally, the expression of Circ_0138960 can reduce proliferation and promote apoptosis, inflammation (increasing levels of TNF-α and IL-6) and oxidative stress in the model of pulpitis using LPS-hDPCs [58]. The molecular mechanism involved in these biological effects included the interaction between Circ_0138960 and miR-545-5p, resulting in decreased expression of this miRNA observed in LPS-hDPCs and samples of human pulpitis. The miR-545-5p can bind the 3′-UTR of MYD88 (MYD88 innate immune signal transduction adaptor) and reduce its expression. Since miR-545-5p is a target of Circ_0138960, the increased expression of this circRNA in pulpitis results in elevated expression of MYD88, which promotes activation of inflammatory pathways. Importantly, the Circ_0138960/miR-545-5p/MYD88 axis is involved in regulation of p65 (RELA proto-oncogene, NF-KB subunit) and IκBα (NFKB inhibitor alpha) phosphorylation, which are important in triggering of the NF-κB pathway. Thus, the increased expression of Circ_0138960 in pulpitis can induce activation of this main inflammatory pathway [58]. Further analyses in more complex models could establish the actual contribution of Circ_0138960 in these biological functions and its potential as a therapeutic target or biomarker since circRNAs show more stability than others RNAs.

### 4.2. CircFKBP5

CircFKBP5 is a circRNA localized in the cytoplasm of hDPSCs. In an in vitro inflammation model, hDPSCs treated with LPS increased apoptosis levels with augmented production of C-casp 3 and Bax and reduced production of Bcl2 (BCL2 apoptosis regulator) and cell viability [59]. Also, LPS-hDPSCs showed enhanced production of IL-1β and TNF-α, diminished capacity of osteogenic/odontogenic differentiation and reduced expression of CircFKBP5 [59]. However, the induced overexpression of CircFKBP5 in LPS-hDPSCs reverted the biological effects described, suggesting that CircFKBP5 can modulate these processes in this model. The molecular mechanism implicated the binding of CircFKBP5 with miR-708-5p. Additionally, this miRNA could bind the 3′-UTR of G protein-coupled receptor (GPCR)-kinase-interacting protein 2 (GIT2) mRNA [59]. Thus, LPS induces reduced expression of CircFKBP5, allowing an increase in the expression of miR-708-5p, which results in the downexpression of the target GIT2, promoting inflammation and apoptosis as well as interfering in the osteogenic/odontogenic differentiation potential of hDPSCs [59]. More studies analyzing the expression modulation of this circRNA could determine its potential in therapeutic strategies. Additionally, validation of CircFKBP5 expression levels in human pulpitis remains to be carried out.

### 4.3. Additional Studies of CircRNAs

Treatment with TNF-α reduces proliferation of hDPSCs and induces the upregulation of 487 circRNAs and downregulation of 708 circRNAs in these cells. The validation showed the increased expression of the circRNAs hsa_circ_0001978, hsa_circ_0003910, hsa_circ_0004314 and hsa_circ_0004417, while the expression of hsa_circ_0035915 was diminished in TNF-α treated hDPSCs [60]. Moreover, the expression of hsa_circ_0001978 and hsa_circ_0004417 increased in samples of human pulpitis. In silico analysis suggested that analyzed circRNAs could regulate the expression of different miRNAs and modulate signaling pathways such as MAPK and WNT [60]. Thus, the inflammatory environment generated in pulpitis can modulate expression of circRNAs in hDPSCs.

On the other hand, the levels of oxidative stress are increased in pulpitis, and it was suggested that oxidative stress causes hDPC death and reduced proliferation of hDPSCs [61,62,63]. In this sense, in an oxidative stress model of hDPSCs treated with hydrogen peroxide, 330 circRNAs showed upregulation and 533 were downregulated in stressed cells as compared with controls. The validation showed an increased expression of hsa_circ_0000257 and reduced expression of hsa_circ_0087354 and hsa_circ_0001946 in stressed cells. It was suggested by bioinformatics analyses that these circRNAs could present different target miRNAs and modulate the oxidative stress environment [64]. The molecular mechanisms to exert the biological effects and potential applications remain to be determined.

## 5. Discussion

The reports analyzed indicated that lncRNAs and circRNAs have emerged as key regulators of diverse biological functions in pulpitis including apoptosis, proliferation, cellular differentiation, oxidative stress, inflammation and immune response. Additionally, in silico analyses have suggested that lncRNAs could modulate processes such as autophagy and ferroptosis in human pulpitis. LncRNAs have been analyzed to a greater extent than circRNAs in the context of pulpitis, and differentially expressed lncRNAs in human pulpitis have been determined. To the best of our knowledge, studies addressing gene expression profiling to identify differentially expressed circRNAs in samples of patients with pulpitis have not been reported yet.

The molecular mechanisms performed by these ncRNAs in the context of pulpitis involved interactions with miRNAs, resulting in the expression modulation of indirect downstream targets and establishing ceRNA regulatory networks in pulpitis. LncRNAs and circRNAs have been shown to exert biological effects through diverse mechanisms, for example, interacting with DNA, binding promoters/enhancers, interacting with proteins and modulating the chromatin structure, binding messenger RNAs and modulating their stability and translation or even interacting with each other [65]. Thus, it is possible that further studies could show a wide variety of molecular mechanisms performed by ncRNAs in pulpitis, which could represent potential therapeutic targets.

The pulp is structured and preserved by different cell types, creating a landscape which is dynamically changing at diverse spatial and temporal levels [66,67]. Expression profiles using single-cell RNA sequencing analysis have shown that the cellular types present in pulp include mesenchymal stem cells, fibroblasts, odontoblasts, endothelial cells, Schwann cells, immune cells, epithelial-like cells and erythrocytes; furthermore, these cell varieties present a diversity of cellular subtypes [68]. To the best of our knowledge, the particular profiles of lncRNA or circRNA expressions in the diverse cell populations present during pulpitis are absent. In this sense, a study was carried out that bioinformatically analyzed the infiltrating immune cells in pulpitis and lncRNAs regulating the expression of inflammatory genes [69]. The report suggested that macrophages and neutrophils are important cells in the development of pulpitis. Additionally, the lncRNAs XIST and KCNQ1OT1 appeared to modulate the expression of relevant cytokines in the biological functions of these infiltrating immune cells [69]; however, this was not determined experimentally. Thus, it is possible that lncRNAs control diverse inflammatory pathways in immune cells during pulpitis. Future studies could determine the expression profiles of lncRNAs in different subpopulations of immune cells as well as other infiltrating or tissue-resident cellular types in pulp. Similarly, the expression profiles of circRNAs in the different immune cell types of pulp have not been addressed. These approaches allow us to appreciate the contribution of ncRNAs in the complex interactions and communications of the diverse cells that regulate the biological functions of pulp in health or disease.

It is interesting that lncRNAs can be packaged and delivered by extracellular vesicles, establishing a system of communication and regulation among cells, and it was suggested that the lncRNA MEG3 can be a cargo molecule in vesicles generated in an in vitro model of pulpitis [39]. Moreover, lncRNA Ankrd26 produced by DPSCs can be delivered inside of extracellular vesicles and taken up by MSCs modulating differentiation [38]. The characterizations of lncRNAs present in extracellular vesicles generated in human pulpitis, experimental pulpitis in animal models or in cellular culture models mimicking the characteristics of pulpitis remain to be determined. CircRNAs can also be found in extracellular vesicles [70]; however, identification, characterization or functional mechanisms of circRNAs packaged into extracellular vesicles have not been addressed in pulpitis. These analyses would allow us to better understand the biological effects of ncRNAs in paracrine signaling during pulpitis and to identify possible therapeutic targets. Moreover, ncRNAs, in association with extracellular vesicles, could represent vehicles to promote differentiation of target cells and to support dental pulp restoration and repair. However, this remains to be established.

Some reported studies (Table 1) showed that through artificial modulation of the lncRNA and circRNA levels (up- or downexpression), different biological effects are generated in the context of pulpitis, suggesting potential applications. However, it is important to consider that these studies were performed in cellular culture models with controlled conditions. Since more complex microenvironments with a diversity of cell types are present in pulpitis, it would be interesting to conduct future studies in animal models to better understand the genuine contribution of lncRNAs and circRNAs in the different biological processes present in pulpitis. Moreover, therapeutic targeting of ncRNAs has been addressed in different diseases [27] and the clinical application represents a significant challenge [71]. The principal problems are related to specificity (undesired on-target outcomes or off-target effects), delivery (including aspects of stability of the molecules, ineffective intracellular transfer or appropriate vehicles for delivery) and tolerability (aspects related to undesirable immune effects) [27]. Despite these, different types of therapeutic nucleic acids targeting ncRNAs have been approved by the FDA (Food and Drug Administration) [27,72]. A deeper knowledge of the contribution of lncRNAs and circRNAs in pulpitis could allow us to address these challenges in future studies.

Correct diagnosis of pulpal status is important to achieve success in treatment; however, current evaluation methods do not accurately or objectively determine the level of pulpal inflammation [73], and molecular approaches could assist in a better assessment. It was suggested that PVT1 identified in the saliva of pulpitis patients could have diagnostic value [50]. Additionally, since extracellular vesicles can be found in biological fluids and circRNAs can be found as cell-free RNAs in fluids as blood, showing high stability [74,75], their identification and characterization in other fluids such as saliva could signify biomarkers or predictors of disease progression; however, this remains to be determined. Since the study of the mechanisms by which lncRNAs and circRNAs modulate pulp inflammation is in its infancy, potential diagnostic applications present many challenges and limitations. For example, identification of a specific circRNA or lncRNA related to pulpitis or associated with a particular stage of pulp inflammation has not been reported. The assessment of the techniques used for detection and quantification of these ncRNAs in pulpitis must show accuracy, reproducibility and sensitivity in clinical settings; however, this has not been addressed. Additionally, the stability of these ncRNAs in fluids such as saliva should be analyzed considering variations in sample collection, processing and storage conditions, which can produce discrepancies in detection of the ncRNAs, affecting accuracy or reproducibility. Thus, the information about the probable uses of lncRNAs and circRNAs as biomarkers or their relevance in therapeutic strategies in pulp inflammation is very limited.

## 6. Conclusions

LncRNAs and circRNAs are differentially expressed in pulpitis. Experimental and in silico studies have shown that these ncRNAs regulate different biological processes in pulpitis such as apoptosis, proliferation, cellular differentiation, oxidative stress, autophagy, ferroptosis, inflammation and immune response.

Cellular culture models mimicking some characteristics of pulpitis have shown that lncRNAs and circRNAs directly interact with different miRNAs, resulting in modulation of the expression of diverse downstream indirect targets and causing various cellular responses. Analysis of these molecular mechanisms and their biological effects in more complex systems as animal models are lacking. Even though lncRNAs and circRNAs show potential as biomarkers or implications in therapeutic strategies, information about the feasibility of the incorporation of these ncRNAs in current diagnostic or therapeutic frameworks is absent. Future studies should clarify these potential applications of lncRNAs and circRNAs in pulpitis.

## Figures and Tables

**Table 1:** Molecular mechanisms analyzed experimentally involving lncRNAs and circRNAs as well as possible biological effects in models resembling some characteristics of pulpitis.

LncRNA	Analyzed Model	Molecular Mechanism	Biological Effects	Ref.
PVT1	Increased expression in hDPCs treated with LPS, as well as in pulp and saliva of patients with pulpitis	PVT1 binds miR-128-3p, resulting in reduced expression of this miRNA	PVT1 promotes inflammation, apoptosis and reduction in cell viability	[36,50]
MEG3	Increased expression in hDPCs treated with LPS and in pulp of patients with pulpitis	The mechanism was not described; however, MEG3 could modulate the p38/MAPK and Wnt/β-catenin signaling pathways	MEG3 promotes inflammation and negatively regulates osteogenic/odontogenic differentiation	[39]
NUTM2A-AS1	Increased expression in hDPCs treated with LPS and in pulp of patients with pulpitis	NUTM2A-AS1 can interact with let-7c-5p and reduce expression of this miRNA, stimulating the expression of HMGB1, since let-7c-5p induces downregulation of HMGB1	NUTM2A-AS1 promotes inflammation and apoptosis	[40]
Ankrd26	Increased expression in rat pulpitis model and expressed in rDPSCs	Ankrd26 binds to miR-150 and reduces miRNA levels, resulting in the increased expression of TLR4, a target of miR-150	Ankrd26 promotes migration and osteogenic differentiation of MSC	[38]
DUXAP8	Increased expression in hDPCs treated with LPS and in pulp of patients with pulpitis	DUXAP8 binds to miR-18b-5p, generating downexpression of this miRNA and increased expression of HIF3A, a target of miR-18b-5p	DUXAP8 promotes inflammation, apoptosis and oxidative stress and negatively regulates cell proliferation	[53]
SNHG7	Decreased expression in a differentiation model using hDPSCs treated with TNF-α	SNHG7 interacts with hsa-miR-6512-3p and reduces the expression of this miRNA	SNHG7 promotes osteogenic/odontogenic differentiation	[41]
TFAP2A-AS1	Increased expression in hDPSCs treated with LPS and in pulp of patients with pulpitis	TFAP2A-AS1 binds mir-32-5p and reduces the expression of this miRNA	TFAP2A-AS1 promotes inflammation and apoptosis and negatively regulates osteogenic/odontogenic differentiation	[56]
LINC00582	Increased expression in pulp of patients with pulpitis. LPS treatment of hDPCs did not modify expression. Analyzed in BALL-1 cell line	The mechanism was not described; LINC00582 does not regulate cell death or inflammation in hDPCs under inflammatory conditions	LINC00582 could regulate inflammatory microenvironment since it promoted migration, proliferation and invasion of a cell line with characteristics of B cells	[42]
FTX	Analyzed in a differentiation model using hDPSCs treated with LPS	Induced overexpression inhibited the expression of the pluripotent transcription factors OCT4, SOX2 and c-MYC	FXT impairs proliferation as well as odontogenic and adipogenic differentiation	[57]
**CircRNA**				
Circ_0138960	Increased expression in hDPCs treated with LPS and in pulp of patients with pulpitis	Circ_0138960 binds miR-545-5p, decreasing expression of this miRNA and increasing expression of MYD88	Circ_0138960 reduces proliferation and promotes apoptosis, inflammation and oxidative stress	[58]
CircFKBP5	Reduced expression in hDPSCs treated with LPS	CircFKBP5 interacts with miR-708-5p, reducing expression and resulting in increased expression of GIT2	CircFKBP5 promotes osteogenic/odontogenic differentiation and negatively regulates apoptosis and inflammation	[59]
